# Supplementary material for: Destabilization of Helix III Initiates Early Serum Amyloid A Misfolding by Exposing Its Amyloidogenic Core
Source: J Phys Chem Lett. 2025 Dec 15;16(51):13116–24. doi: 10.1021/acs.jpclett.5c03467 (PMC12746452; doi:10.1021/acs.jpclett.5c03467)
Supplement: Supplementary file 1 [file jz5c03467_si_001.pdf]

Destabilization of Helix III Initiates Early Serum Amyloid A Misfolding by Exposing Its Amyloidogenic Core

Haidara Nadwa,† Z. Faidon Brotzakis,*,‡,¶ Annalisa Santucci,† Daniela Braconi,† and Michele Vendruscolo*,¶

†*Dipartimento di Biotecnologie, Chimica e Farmacia, Università degli Studi di Siena, via Aldo Moro 2, 53100 Siena, Italy*

‡*Institute for Bioinnovation, Biomedical Sciences Research Center "Alexander Fleming", 34 Fleming Street, 16672, Vari, Greece*

¶*Centre for Misfolding Diseases, Department of Chemistry, University of Cambridge, Lensfield Rd. CB21EW, Cambridge, UK*

E-mail: brotzakis@fleming.gr; mv245@cam.ac.uk

Devising Collective Variables

(a)

(b)

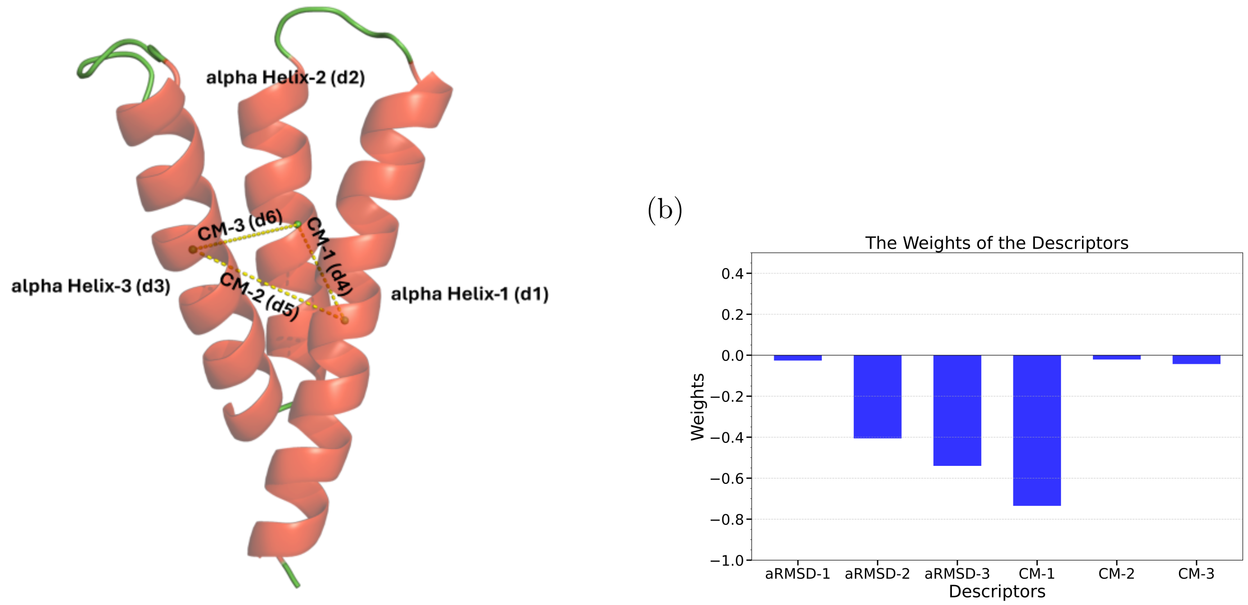

Figure S1: (a) Cartoon structure of SAA_{1-76} with the three α-helices shown in red; the distances between the centers of mass of the α-helices used as descriptors are also indicated. (b) Weights assigned by HLDA to each of the descriptors.

The Efficiency of the Simulation

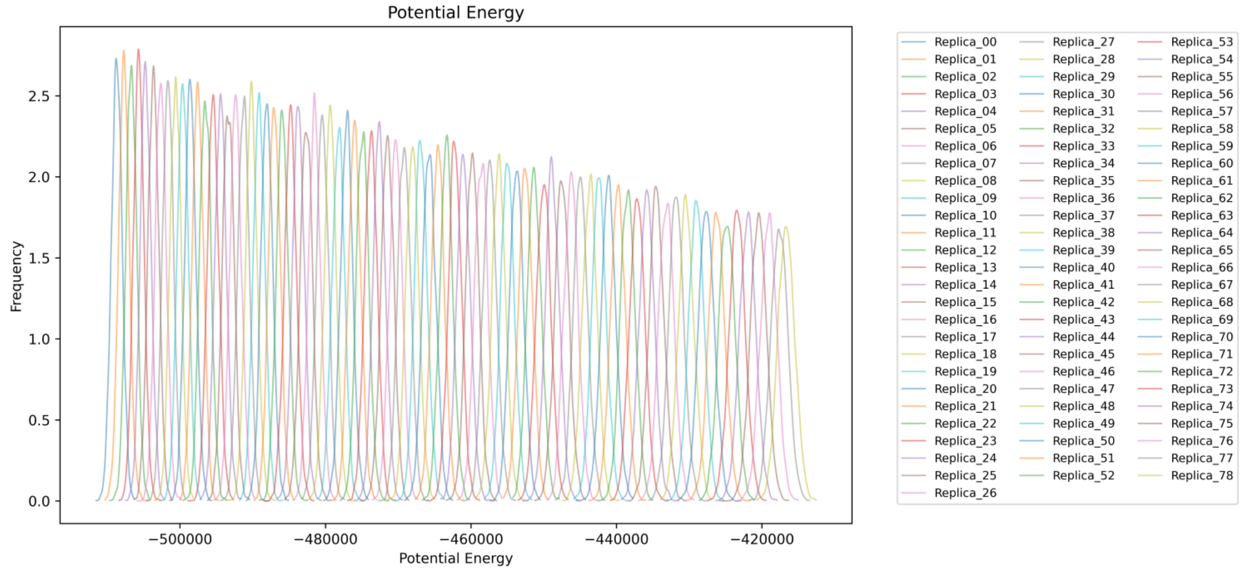

Figure S2: Potential energy probability distributions at all simulated temperatures

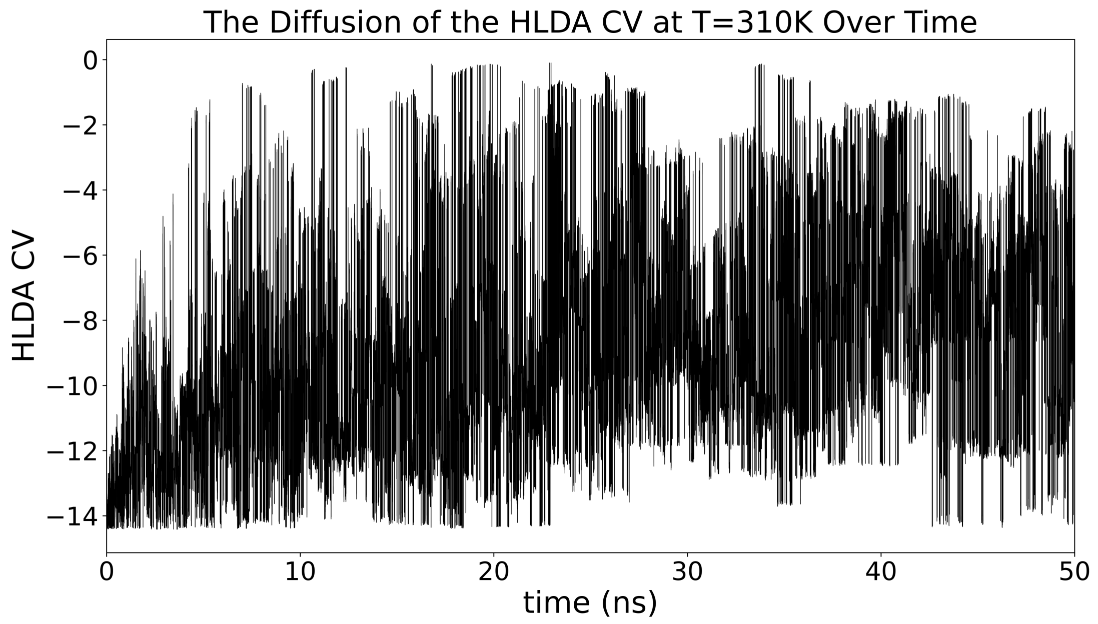

Figure S3: HLDA CV time dependence taken from T=310K replica of PTMetaD simulations.

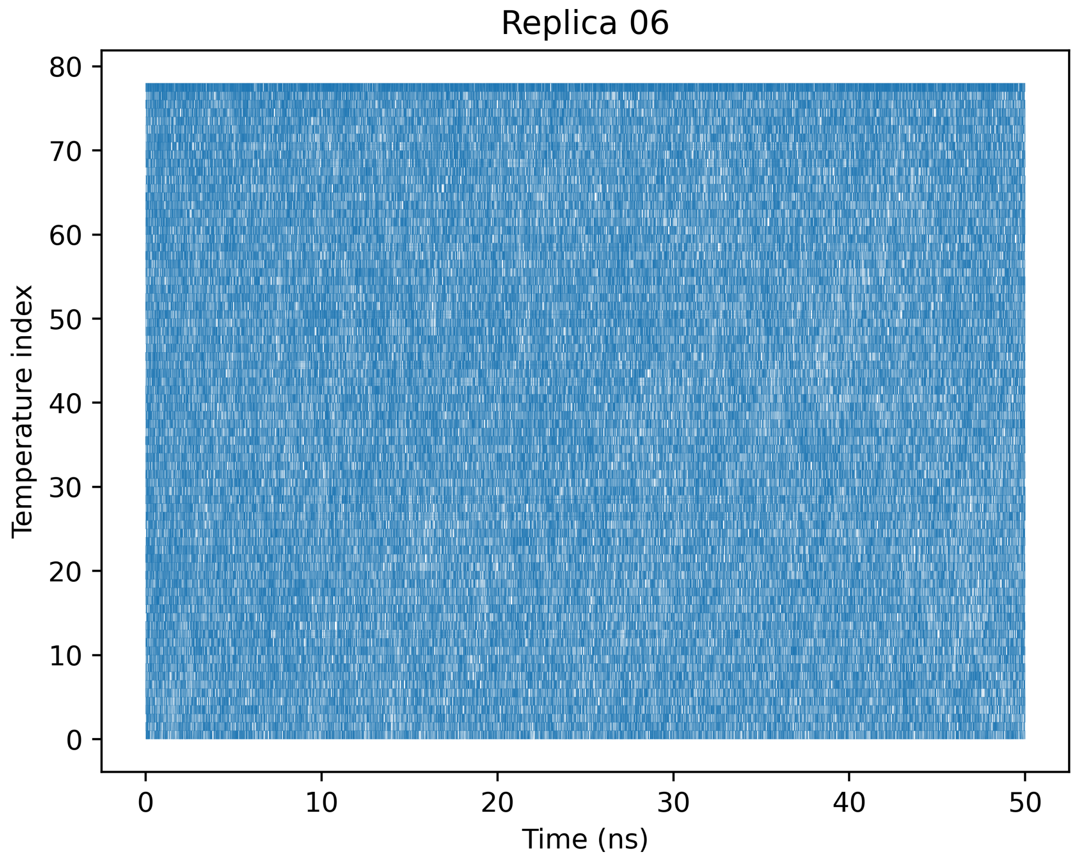

Figure S4: The time evolution of replica 6 through temperature space

The Convergence of the Simulation

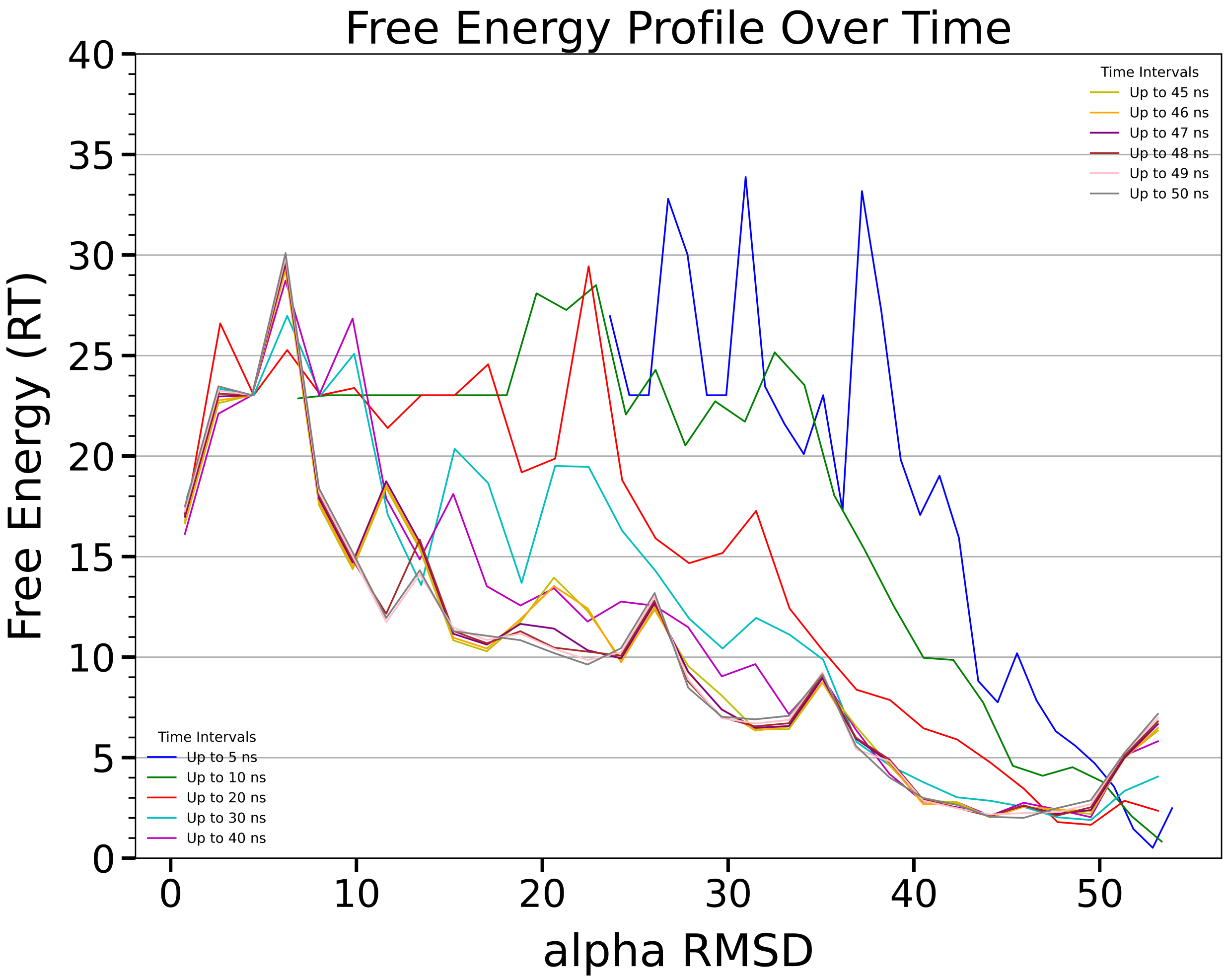

Figure S5: Overlapping of free energy profiles along αRMSD CV in different time windows

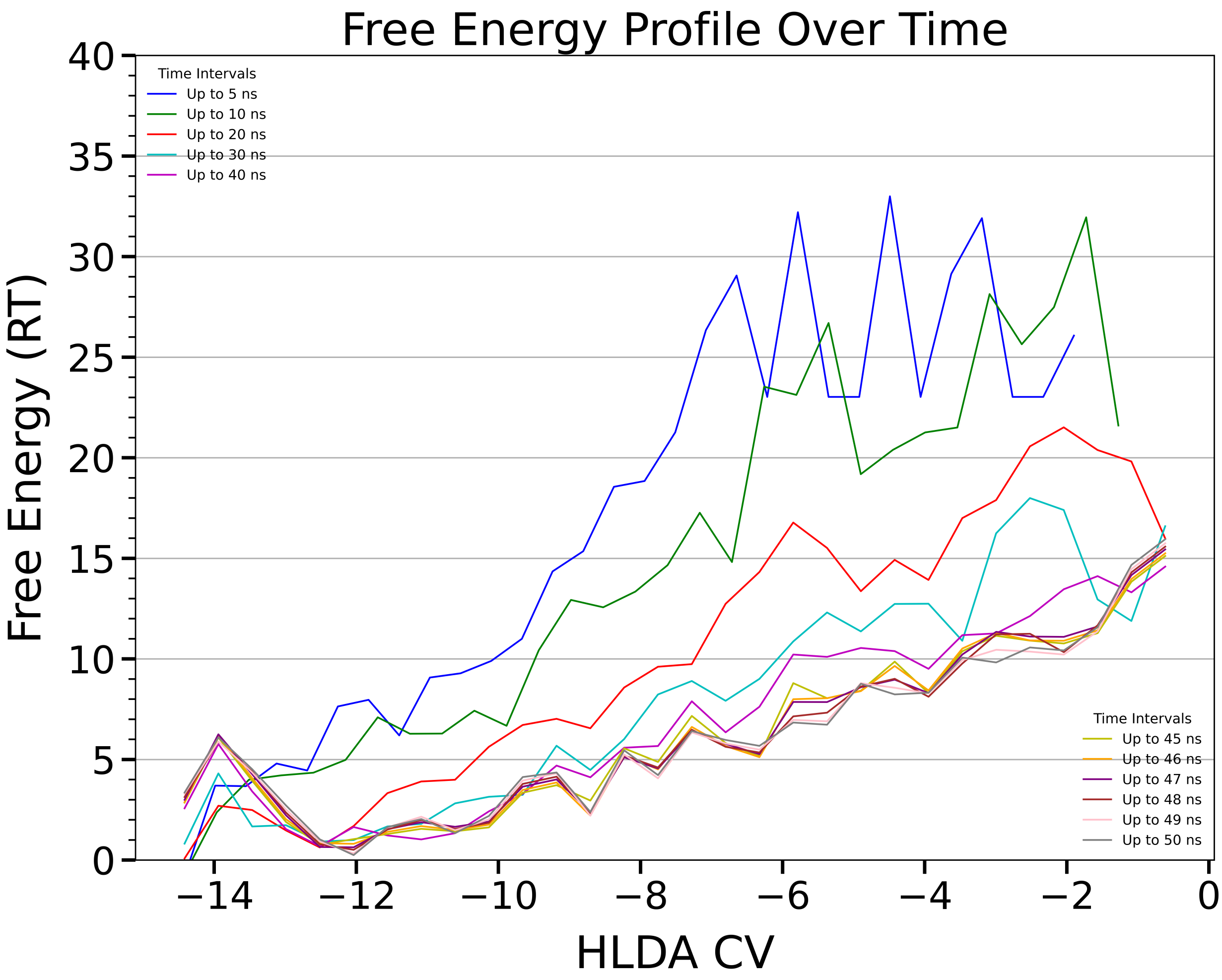

Figure S6: Overlapping of free energy profiles along HLDA CV in different time windows

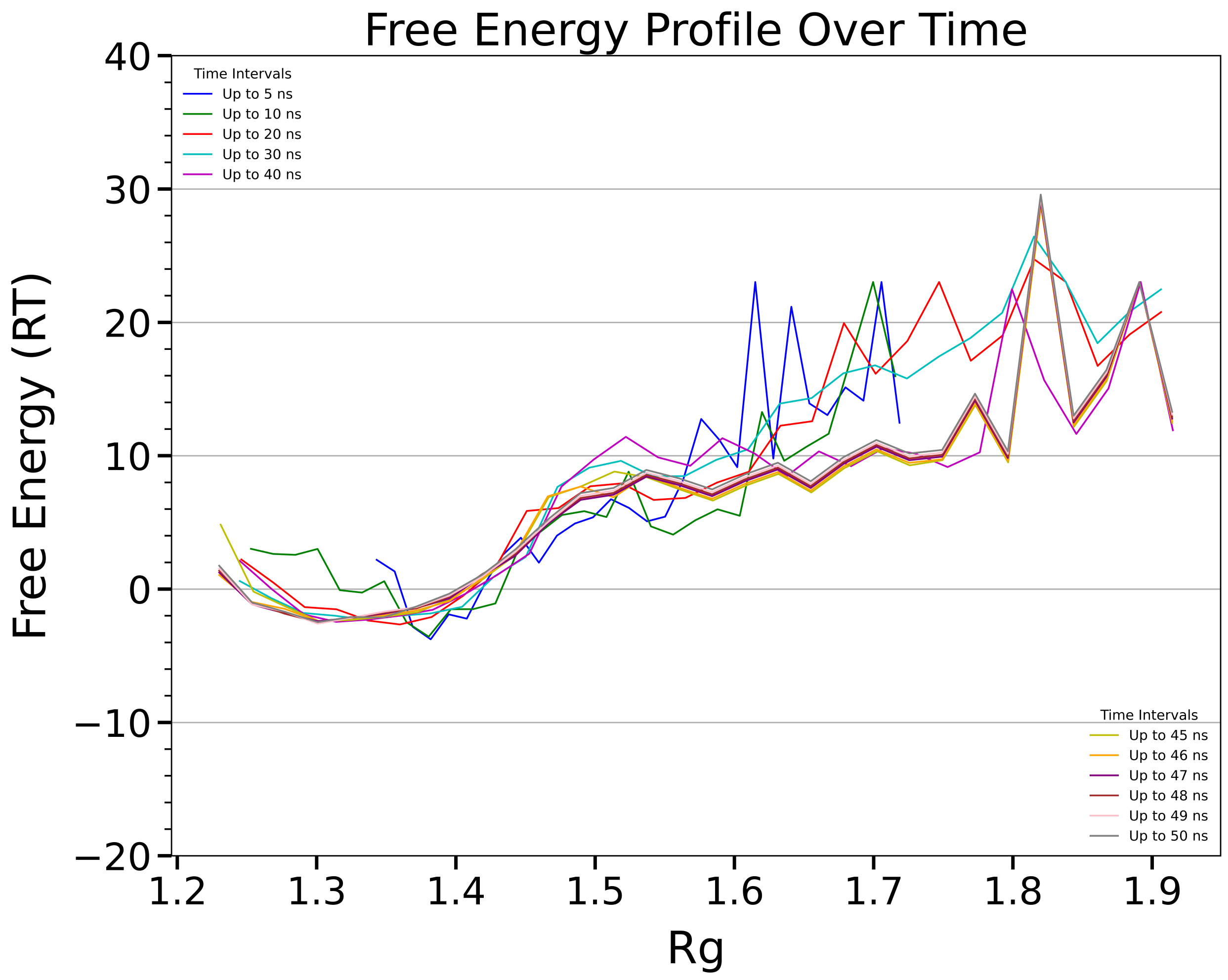

Figure S7: Overlapping of free energy profiles along Rg CV in different time windows

SASA Analysis along the identified misfolding pathways

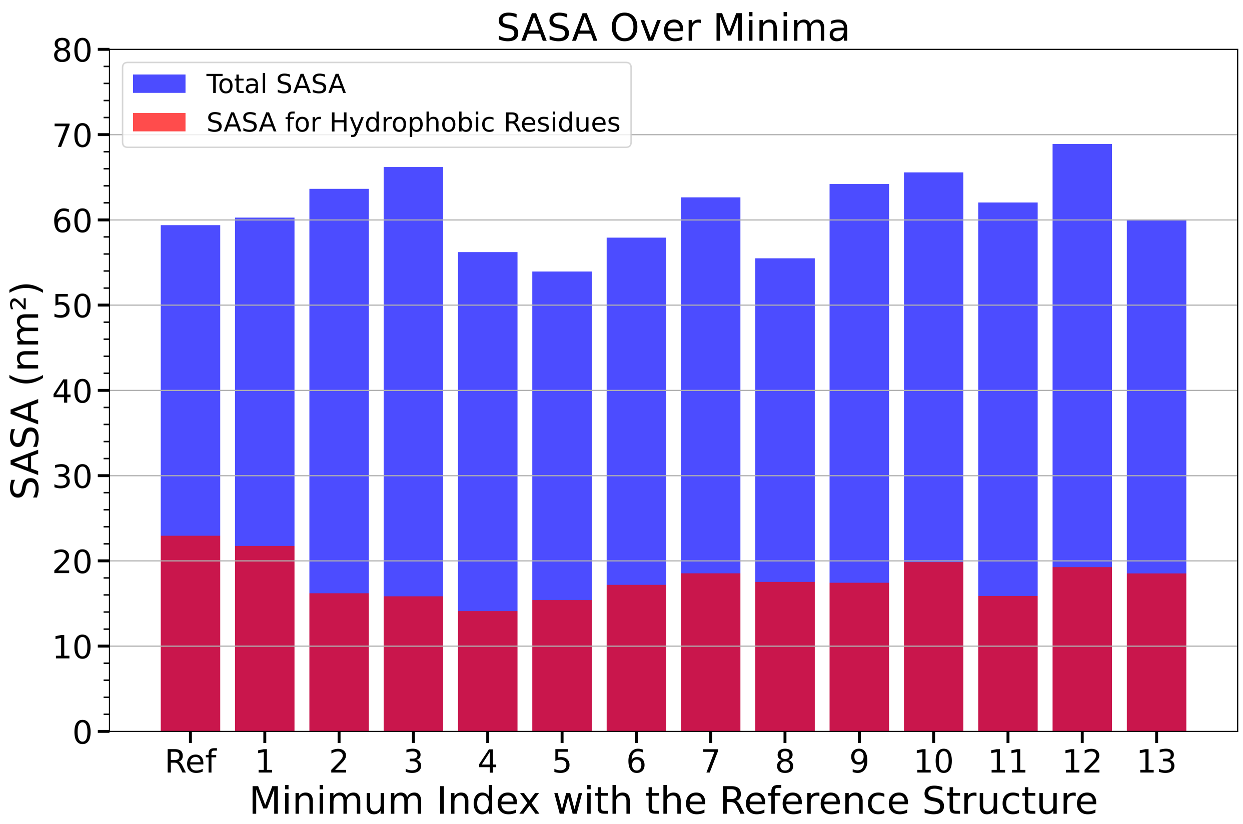

Figure S8: Total SASA for the protein and for the hydrophobic residues for the representative structure sampled during the simulations along aRMSD and HLDA-CV

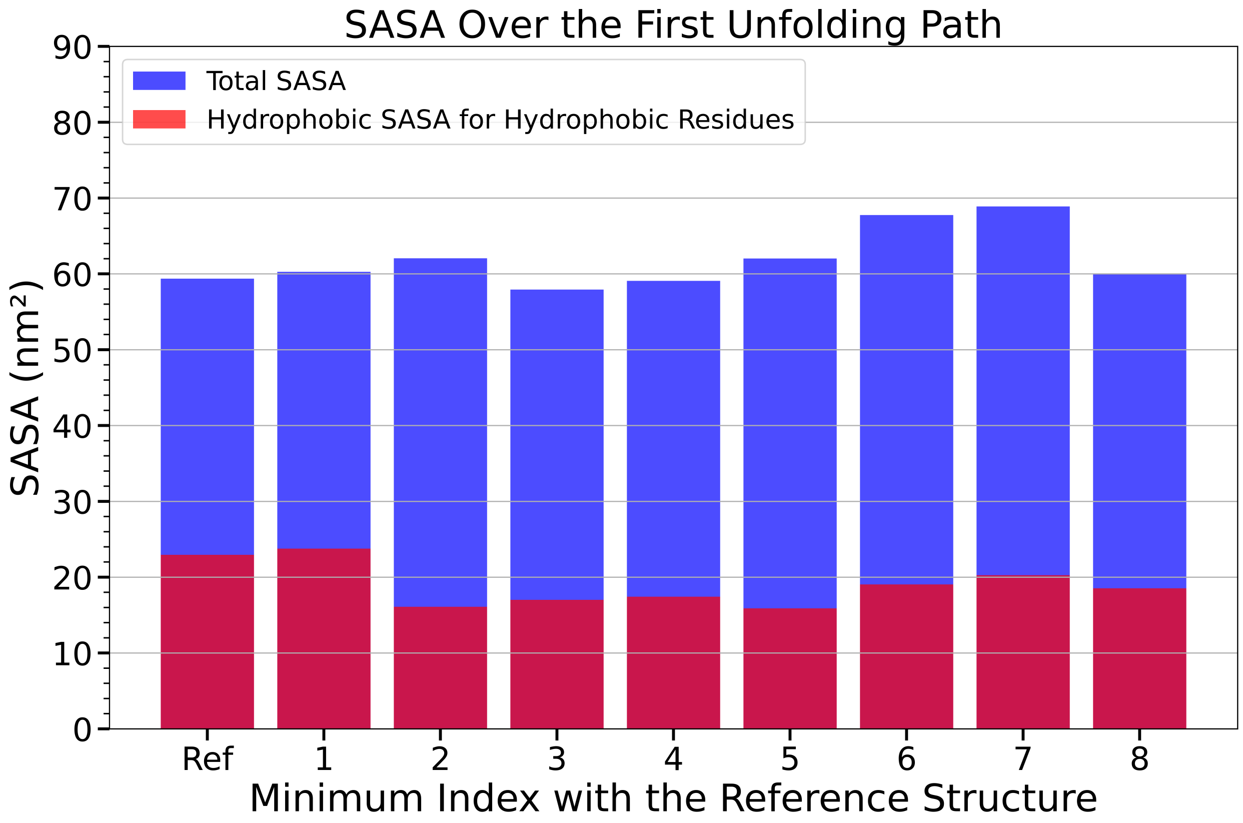

Figure S9: Total SASA for the protein and for the hydrophobic residues for the representative structure sampled during the simulations along the first misfolding path along aRMSD and Rg

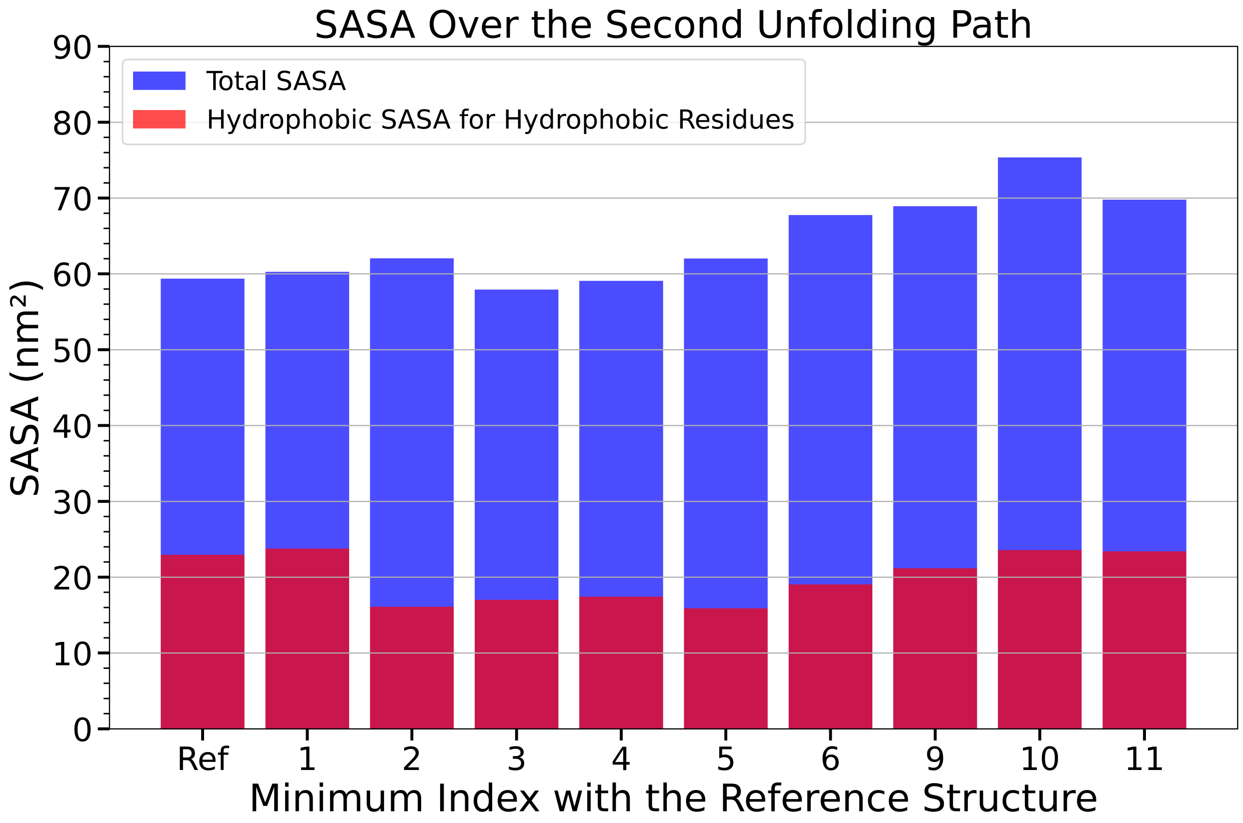

Figure S10: Total SASA for the protein and for the hydrophobic residues for the representative structure sampled during the simulations along the second misfolding path along aRMSD and Rg

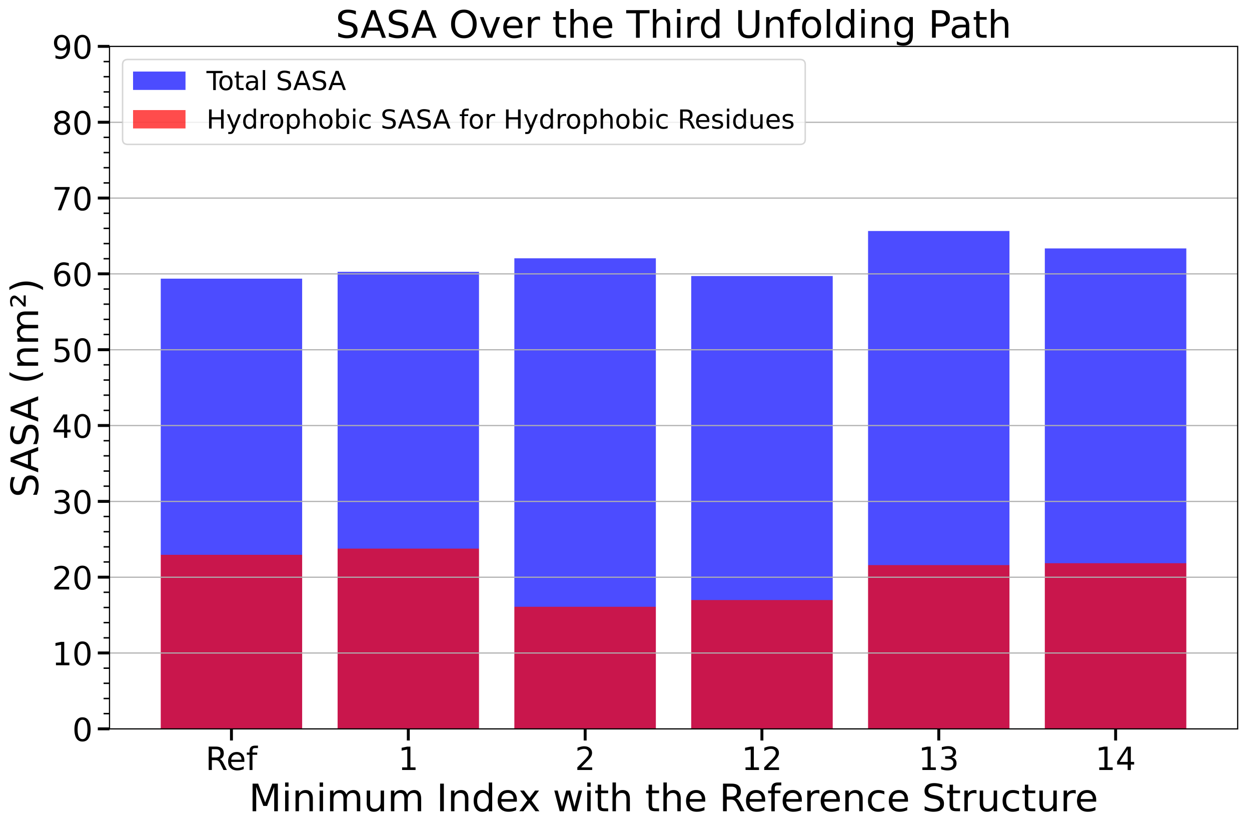

Figure S11: Total SASA for the protein and for the hydrophobic residues for the representative structure sampled during the simulations along the third misfolding path along aRMSD and Rg
